# Severe maternal morbidity: admission shift from intensive care unit to obstetric high-dependency unit

**DOI:** 10.1186/s12884-022-04480-x

**Published:** 2022-02-21

**Authors:** Ning Gu, Yaning Zheng, Yimin Dai

**Affiliations:** Department of Obstetrics and Gynecology, Nanjing University Medical School Affiliated Nanjing Drum Tower Hospital, 321 Zhongshan Road, Nanjing, 210008 China

**Keywords:** Severe maternal morbidity, Intensive care unit, High dependency unit, Hypertensive disorders in pregnancy, Pre-existing medical disease

## Abstract

**Background:**

To study temporal trends of intensive care unit (ICU) admission in obstetric population after the introduction of obstetric high-dependency unit (HDU).

**Methods:**

This is a retrospective study of consecutive obstetric patients admitted to the ICU/HDU in a provincial referral center in China from January 2014 to December 2019. The collected information included maternal demographic characteristics, indications for ICU and HDU admission, the length of ICU stay, the total length of in-hospital stay and APACHE II score. Chi-square and ANOVA tests were used to determine statistical significance. The temporal changes were assessed with chi-square test for linear trend.

**Results:**

A total of 40,412 women delivered and 447 (1.11%) women were admitted to ICU in this 6-year period. The rate of ICU admission peaked at 1.59% in 2016 and then dropped to 0.67% in 2019 with the introduction of obstetric HDU. The average APACHE II score increased significantly from 6.8 to 12.3 (P < 0.001) and the average length of ICU stay increased from 1.7 to 7.1 days (P < 0.001). The main indications for maternal ICU admissions were hypertensive disorders in pregnancy (39.8%), cardiac diseases (24.8%), and other medical disorders (21.5%); while the most common reasons for referring to HDU were hypertensive disorders of pregnancy (46.5%) and obstetric hemorrhage (43.0%). The establishment of HDU led to 20% reduction in ICU admission, which was mainly related to obstetric indications.

**Conclusions:**

The introduction of HDU helps to reduce ICU utilization in obstetric population.

## Background

Admission of pregnant or postpartum women to intensive care unit (ICU) is an indicator of severe maternal morbidity and an endpoint for clinical audit in quality of care [1]. Despite the proportion of high-risk pregnancies in China increased from 15.7 to 24.7%, maternal mortality rates decreased from 34.2 per 100,000 to 18.3 per 100,000 live births from 2008 to 2018 [2], which implied an improvement in perinatal care. On the other hand, the growth of complicated pregnancies caused an increase in maternal ICU admissions [3], which meant a rising need of medical resources and expenses. Therefore, monitoring of the trend of ICU admission may help us understand the reasons for the change and develop interventions to improve medical service.

High dependency unit (HDU) provides a level of care which lies in between a general ward and an ICU, which has been suggested to reduce the need for ICU beds [4]. As previous researches from China showed, the incidence of pregnancy related ICU admission varied from 0.56 to 1.6% [3, 5]. However, little was known about the respective roles of obstetric HDU and ICU in caring severe maternal morbidity in this region. Accordingly, the present study aims to study the temporal trend of ICU admission with the introduction of maternal HDU and assess the characteristics of pregnancy-related ICU and HDU admissions.

## Methods

### Study design and settings

This is a retrospective study of consecutive obstetric patients admitted to the ICU/HDU of Nanjing Drum Tower Hospital from January 2014 to December 2019. The obstetric service in this hospital has high patient acuity and was accredited as a provincial referral center for severe maternal morbidity in 2016. To cope with the rising demand for a higher level of care, the institute introduced obstetric HDU in 2017. The general ICU was a 20-bed unit led by intensivists, whereas the obstetric HDU was a 5-bed unit staffed with obstetricians and specialized nurses.

The research has been approved by the Ethical Committee of Nanjing Drum Tower Hospital and a waiver of individual informed consent was granted. Information of cases admitted to ICU of any causes during the pregnancy or within 42 days postpartum was abstracted from medical records and did not identify participants. Women delivered in other institutions and transferred to the hospital in postpartum period were excluded in the analysis.

### Participants and Variables

The women’s characteristics included age, parity, type of pregnancy (singleton or multiple), gestational age of delivery and mode of delivery (vaginal or cesarean delivery). The ICU admission information included the length of ICU stay, the total length of in-hospital stay and APACHE II score [6], calculated within 24 h of ICU admission based on the worst clinical, and physiologic indicators. The indications for ICU/HDU admissions were classified into obstetric and non-obstetric complications. Organ dysfunction was defined using the WHO Working Group approach [7], which included clinical criteria, laboratory markers and management-based proxies. If a woman was admitted to the ICU more than once, the stay with the worst clinical manifestations (the highest APACHE II score or the longest stay) were included in this study.

### Statistical methods

Statistical analysis was performed with SPSS 26.0. Normally distributed data were presented as mean with SD; and categorical outcomes were summarized using frequency distributions. Chi-square and ANOVA tests were used to determine statistical significance. The temporal changes were assessed with chi-square test for linear trend.

To estimate the associations between maternal clinical features and ICU admission, characteristics (age, obesity, multiple pregnancy, pre-gestational hypertension, pre-gestational diabetes, pre-eclampsia, and placenta previa) for women with and without ICU admission were compared using Chi-square tests, odds ratios (OR) and 95% confidence intervals (CI). Multiple logistic regression was used to estimate OR of ICU admission while adjusting for maternal risk factors and the introduction of HDU. A p value of 0.05 was used as the cut-point for significance.

## Results

During this 6-year period, a total of 40,412 women delivered at the hospital and 447 women were admitted to ICU, making an overall ICU admission rate of 1.11%. The rate peaked at 1.59% in 2016 and then dropped to 0.67% in 2019 (Table 1).

There were 9 maternal deaths and the maternal mortality rates were 2.0 per 100 ICU admissions and 22.3 per 100,000 hospital deliveries. No significant differences were found regarding the yearly ICU mortality rates. Cardiac diseases were the leading cause of maternal death (5/9, including 3 cases of severe pulmonary hypertension, one case of heart failure secondary to Sjogren syndrome and one woman with prosthetic valve thrombosis), followed by infections (2/9, one with severe virus pneumonia and one with septic miscarriage), lymphoma (1/9) and cerebral hemorrhage (1/9).

### Demographics of Pregnancy-related ICU admission

Overall, the mean age of the women admitted to the ICUs was 29.4 years; 16.8% were 35 years of age or older (Table 1). Nulliparous women comprised 65.1% of the ICU admissions, and the proportion decreased over time (P < 0.001). Most of the patients were admitted during the postpartum period (88.1%) and had undergone cesarean deliveries (85.7%).

**Table 1:** Characteristics of women with pregnancy-related ICU admissions

	Overall	2014	2015	2016	2017	2018	2019	P
ICU admission	n = 447	n = 52	n = 56	n = 117	n = 102	n = 70	n = 50	
No. of deliveries	40,412	5737	5269	7357	7428	7107	7514	
Rate of ICU admission (%)	1.11	0.91	1.06	1.59	1.37	0.98	0.67	<0.001
Age (mean ± sd)	29.4 ± 5.3	29.1 ± 5.3	29.1 ± 5.7	29.1 ± 4.9	29.6 ± 4.8	29.6 ± 5.8	30.3 ± 5.7	0.818
Age ≥ 35 years n (%)	75 (16.8)	7 (13.5)	12 (21.4)	17 (14.5)	17(16.7)	11 (15.7)	11 (22.0)	0.545
Nulliparous n (%)	291 (65.1)	47 (90.4)	47 (83.9)	71 (60.7)	59 (57.8)	37 (52.9)	30 (60.0)	<0.001
Multiple pregnancy n (%)	39 (8.7)	5 (9.6)	2 (3.6)	12 (10.3)	11(10.8)	5 (7.1)	4 (8.1)	0.691
Pre-gestational BMI ≥28 kg/m^2^ n (%)	25 (5.6)	2 (3.8)	5 (8.9)	3 (2.6)	4 (3.9)	6 (8.6)	5 (10.0)	0.209
GA at delivery^a^								0.145
<28 wks n (%)	63 (14.2)	5 (9.6)	14 (25.0)	12 (10.3)	13 (12.7)	8 (11.6)	11 (22.0)	
28-31^+6^ wks n (%)	86 (19.3)	8 (15.4)	11 (19.6)	21 (18.1)	24 (23.5)	11 (15.9)	11 (22.0)	
32-36^+6^ wks n (%)	174 (39.1)	17 (32.7)	16 (28.6)	51 (44.0)	40 (39.2)	32 (46.4)	18 (36.0)	
≥37 wks n (%)	122 (27.4)	22 (42.3)	15 (26.8)	32 (27.6)	25 (24.5)	18 (26.1)	10 (20.4)	
Cesarean deliveries n (%)	383 (85.7)	45 (86.5)	45 (80.4)	104 (88.9)	89 (87.3)	60 (85.7)	40 (80.0)	0.662
Postpartum admission n (%)	394 (88.1)	49 (94.2)	52 (92.9)	108 (92.3)	94(92.2)	57 (81.4)	34 (68.0)	<0.001
APACHE II score (mean ± sd)	10.5 ± 5.6	6.8 ± 3.6	9.4 ± 8.1	11.3 ± 5.0	10.9 ± 4.8	10.9 ± 5.3	12.3 ± 5.2	<0.001
Length of ICU stay (d) (mean ± sd)	4.5 ± 6.6	1.7 ± 1.6	3.3 ± 4.5	3.7 ± 4.2	5.2 ± 8.6	5.9 ± 7.4	7.1 ± 8.5	<0.001
In-hospital stay (d) (mean ± sd)	11.7 ± 8.8	8.4 ± 3.6	10.4 ± 6.9	10.8 ± 6.9	12.5 ± 10.1	13.8 ± 11.5	14.1 ± 9.8	0.002
ICU mortality rate n (%)	9 (1.9)	0 (0)	1(1.8)	2 (1.7)	3 (2.9)	1 (1.4)	2 (4.0)	0.802

^a^ Two cases of maternal death before delivery in 2016 and 2018 respectively

The average APACHE II score, an indicator of severity, increased significantly from 6.8 in 2014 to 12.3 in 2019 (P < 0.001). At the same time, the average length of ICU stay increased from 1.7 day to 7.1 days (P < 0.001), and the mean hospital stay increased from 8.4 to 14.1 days (P = 0.002).

### Indications for maternal ICU admission

The main causes for maternal ICU admission were hypertensive disorders in pregnancy (39.8%), cardiac diseases (24.8%), and other medical disorders (21.5%) (Table 2). Women could have had more than one complication. Over the 6-year period, the proportion of obstetric hemorrhage (from 25.0 to 4.0%; P = 0.015) and hypertensive disorders (from 46.2 to 28.0%; P = 0.034) decreased significantly, whereas the percentage of cardiac diseases increased from 26.9 to 42.0% (P = 0.024).

**Table 2:** Indications for Pregnancy-Related ICU Admissions^a^

	Overall	2014	2015	2016	2017	2018	2019	P trend
No. of ICU admission	447	52	56	117	102	70	50	
Obstetric Indications								
Hemorrhage	56 (12.5)	13 (25.0)	3 (5.4)	18 (15.4)	13 (12.7)	7 (10.0)	2 (4.0)	0.015
Hypertensive disorders	178 (39.8)	24 (46.2)	26 (46.4)	45 (38.5)	46 (45.1)	23 (32.9)	14 (28.0)	0.034
Obstetric infections	11 (2.5)	1 (1.9)	3 (5.4)	2 (1.7)	2 (2.0)	3 (4.3)	0 (0)	0.609
Amniotic fluid embolism	4 (0.9)	2 (3.8)	0 (0)	1 (0.9)	0 (0)	0 (0)	1 (2.0)	0.319
Acute fatty liver of pregnancy	17 (3.8)	2 (3.8)	0 (0)	5 (4.3)	6 (5.9)	3 (4.3)	1 (2.0)	0.740
Other obstetric complications	6 (1.3)	1 (1.9)	2 (3.6)	1 (0.9)	1 (1.0)	1 (1.4)	0 (0)	0.272
Non-obstetric Indications								
Cardiac diseases	111 (24.8)	14 (26.9)	12 (21.4)	20 (17.1)	23 (22.5)	21 (30.0)	21 (42.0)	0.024
Non-obstetric infections	16 (3.6)	0 (0)	1 (1.8)	3 (2.6)	6 (5.9)	4 (5.7)	2 (4.0)	0.071
Pancreatitis	26 (5.8)	0 (0)	2 (3.6)	7 (6.0)	7 (6.9)	5 (7.1)	5 (10.0)	0.024
Other medical disorders	96 (21.5)	16 (30.8)	12 (21.4)	26 (22.2)	20 (19.6)	9 (12.9)	13 (26.0)	0.190
Organ Dysfunctions								
Cardiovascular dysfunction	83 (18.6)	3 (5.8)	5 (8.9)	14 (12.0)	23 (22.5)	22 (31.4)	16 (32.0)	<0.001
Respiratory dysfunction	130 (29.1)	6 (11.5)	16 (28.6)	40 (34.2)	24 (23.5)	22 (31.4)	22 (44.0)	0.007
Renal dysfunction	40 (8.9)	0 (0)	7 (12.5)	8 (6.8)	15 (14.7)	5 (7.1)	5 (10.0)	0.170
Hematological dysfunction	112 (25.1)	12 (23.1)	14 (25.0)	31 (2.6)	26 (25.5)	14 (20.0)	15 (30.0)	0.832
Hepatic dysfunction	17 (3.8)	2 (3.8)	1 (1.8)	7 (6.0)	6 (5.9)	0 (0)	1 (2.0)	0.420
Neurological dysfunction	20 (4.5)	0 (0)	3 (5.4)	6 (5.1)	5 (4.9)	1 (1.4)	5 (10.0)	0.182
Uterine dysfunction	13 (2.9)	2 (3.8)	2 (3.6)	5 (4.3)	1 (1.0)	2 (2.9)	1 (2.0)	0.366

^a^Patients can have more than one indication

In the analysis of the breakdown of maternal organ dysfunctions (Table 2), respiratory dysfunction (29.1%) had the highest incidence, followed by hematological dysfunction (25.1%) and cardiovascular dysfunction (18.6%). From 2014 to 2019, both respiratory dysfunction rates (11.5 - 44.0%, P = 0.007) and cardiovascular dysfunction rates (5.8 - 32.0%, P < 0.001) showed increasing trends.

### Decrease in ICU admission after introduction of HDU

After the introduction of HDU, the rate of ICU admission reduced by 18.0% (1.23% in 2014-16 vs. 1.01% in 2017-19), and HDU admission (1575 cases) amounted to 7.15% of total obstetric population. The obstetric indications for HDU admissions were hypertensive disorders of pregnancy (733, 46.5%), obstetric hemorrhage (678, 43.0%), other pregnancy complications (16, 1.0%), and obstetric infections (2, 0.1%). Heart diseases (74, 4.7%), other medical disorders (161, 10.2%), pancreatitis (6, 0.4%), and non-obstetric infections (3, 0.2%) accounted for the non-obstetric causes. The cause-specific rates of pregnancy-related ICU admissions significantly decreased for hypertensive disorders (OR = 0.573, 95% CI =0.424-0.775) and obstetric hemorrhage (OR = 0.518, 95% CI =0.302-0.888) in 2017-2019 (Table 3).

**Table 3:** Indications for HDU and ICU admissions before and after establishment of HDU^a^

	2014-2016		2017-2019				OR (95%CI) ^b^
	n	ICU admission n (%)	n	ICU and HDU admission n (%)	HDU admission n (%)	ICU admission n (%)	
Obstetric hemorrhage	2539	34 (1.3)	3151	700 (22.2)	678 (21.5)	22 (0.7)	0.518 (0.302-0.888)
Hypertensive disorders	1468	95 (6.5)	2176	816 (37.5)	733 (33.7)	83 (3.8)	0.573 (0.424-0.775)
Obstetric infections	207	6 (2.9)	333	7 (2.1)	2 (0.6)	5 (1.5)	0.511 (0.154-1.695)
Cardiac diseases	263	46 (17.5)	403	139 (27.6)	74 (18.4)	65 (16.1)	0.907 (0.599-1.373)
Non-obstetric infections	25	4 (16.0)	37	15 (40.5)	3 (8.1)	12 (32.4)	2.520 (0.707-8.988)
Pancreatitis	16	9 (56.3)	31	23 (74.2)	6 (19.4)	17 (54.8)	0.944 (0.280-3.183)

^a^Patients can have more than one indication. ^b^ Odds ratios for indication-specific ICU admission rates before and after establishment of HDU

### Contribution of maternal risk factors to ICU admission

In the obstetric population, the incidence of maternal age ≥ 35 years (10.5 - 14.3%, P < 0.001), pre-gestational BMI ≥ 28 kg/m^2^ (0.4 - 3.5%, P < 0.001), multiple pregnancies (3.9 - 5.0%, P < 0.001), pre-gestational hypertension (1.2 - 1.8%, P = 0.001) and preeclampsia (4.9 - 6.3%, P < 0.001) increased significantly from 2014 to 2019 (Table 4).

**Table 4:** Temporal Trends in the proportion of women with high-risk pregnancies^a^

	2014	2015	2016	2017	2018	2019	P trend
No. of deliveries	5737	5269	7357	7428	7107	7514	
Maternal age ≥ 35 years	603 (10.5)	741 (14.1)	1068 (14.5)	1387 (18.7)	1119 (15.7)	1071 (14.3)	<0.001
Multiple pregnancies	224 (3.9)	282 (5.4)	342 (4.6)	413 (5.6)	442 (6.2)	377 (5.0)	<0.001
Pre-gestational BMI ≥ 28 kg/m^2^	24 (0.4)	110 (2.1)	140 (1.9)	134 (1.8)	187 (2.6)	265 (3.5)	<0.001
Pre-gestational hypertension	71 (1.2)	72 (1.4)	119 (1.6)	142 (1.9)	128 (1.8)	138 (1.8)	0.001
Pre-gestational diabetes	92 (1.6)	62 (1.2)	76 (1.0)	71 (1.0)	90 (1.3)	117 (1.6)	0.768
Placenta previa	115 (2.0)	155 (2.9)	176 (2.4)	211 (2.8)	193 (2.7)	172 (2.3)	0.519
Gestational diabetes	1107 (19.3)	527 (10.0)	779 (10.6)	930 (12.5)	763 (10.7)	798 (10.6)	<0.001
Preeclampsia	280 (4.9)	279 (5.3)	444 (6.0)	468 (6.3)	437 (6.1)	475 (6.3)	<0.001

^a^Values are given as number (percentage)

Associations between maternal risk factors and ICU admission are included in Table 5. Women with pre-eclampsia were found to have the highest adjusted odds of ICU admission (aOR =7.752, 95% CI = 6.211-9.709), followed by placenta previa (aOR =3.571, 95% CI = 2.463-5.181) and pre-gestational diabetes (aOR =2.538, 95% CI =1.656-3.891). After adjusting for the maternal risk factors, the establishment of HDU was related to 20% reduction of ICU admission (aOR =0.796, 95% CI =0.658-0.962). Pre-gestational obesity, multiple pregnancy and pre-gestational hypertension were not independent risk factors for ICU admission.

**Table 5:** Maternal risk factors for ICU admission

	Crude OR (95% CI)	Adjusted OR (95% CI)^a^
Maternal age ≥ 35 years	1.161 (0.904-1.490)	–
Multiple birth	1.776 (1.275-2.474)	1.211 (0.860-1.706)
Pre-gestational BMI ≥28 kg/m^2^	2.776 (1.844-4.180)	1.073 (0.676-1.704)
Pre-gestational hypertension	4.459 (2.952-6.736)	0.977(0.621-1.536)
Pre-gestational diabetes	4.731 (3.154-7.049)	2.538 (1.656-3.891)
Placenta previa	2.944 (2.044-4.241)	3.571 (2.463-5.181)
Gestational diabetes	0.790 (0.578-1.081)	–
Preeclampsia	8.002 (6.540-9.791)	7.752 (6.211-9.709)
Establishment of HDU	0.820 (0.680-0.988)	0.796 (0.658-0.962)

^a^Odds ratios were adjusted for multiple birth, pre-gestational BMI ≥28 kg/m^2^, pre-gestational hypertension, pre-gestational diabetes, placenta previa, preeclampsia and establishment of HDU

## Discussion

This is the first study analyzing HDU in management of severe maternal morbidity in China. In this study from a large referral center in China, the ICU admission rate decreased by 20%, whereas the severity of the cases increased after introduction of obstetric HDU. Nonetheless, the maternal mortality rate remained stable. Hypertensive disorders in pregnancy was the most common cause for ICU and HDU admission, while cardiac diseases and hemorrhage accounted for the second largest proportion in ICU and HDU cases respectively.

The research showed a rising trend of high-risk pregnancies in our population, which resulted in an increased demand for higher level of care. The rate of HDU admission in this study was 7.1%, which was higher than the UK survey (4.2%) [8] and lower than two single center reports from India (11.1 and 11.2%) [9, 10]. The variations were likely to be influenced by annual birth rate, characteristics of the population, and criteria for transferring women to ICU. Similar to the literatures [9, 10], our research showed the main indications for high dependency care were obstetric reasons and the introduction of HDU caused a significate reduction in obstetric hemorrhage and hypertensive disorders related ICU admission, while women with severe medical co-morbidities and women with organ dysfunction still needed ICU care. It is notable that our obstetric HDU has a relatively large capacity and situated in the obstetric wards rather than close to the ICU; therefore, high-risk patients who would otherwise have been managed in general ward were managed in HDU. For example, patients requiring intravenous antihypertensive drugs or close monitoring of input and output now received specialized care by the HDU team. Whether obstetric HDU can improve patient outcomes remained uncertain [11]. In our practices, doctors and nurses believed that the integration of intermediate care in obstetric practice benefited patient safety and reduced work stress.

ICU provides invasive monitoring and organ support to women with severe morbidity. Since we triaged women towards different level of units, the case severity of ICU admission increased significantly, as we showed a rising trend in the APACHE II score and length of stay among ICU cases. Consistent with other studies [3, 5, 12], cardiac diseases and other medical disorders accounted for the majority of non-obstetric indications in our ICU admissions and often required invasive hemodynamic monitoring, mechanical ventilation and multidisciplinary management. We also noticed that women with non-obstetric indications were more complicated than those with obstetric indications. Eight out of nine maternal mortalities in our cohort were due to non-obstetric causes and the proportion of medical disorder indicated ICU admissions remained unchanged after HDU introduction.

In addition to triage of less severe patients to HDU, the decrease in obstetric hemorrhage related ICU admission was also attributable to our institutional quality improvement project targeted to prevent complications in severe postpartum hemorrhage [13]. After the introduction of a criteria-based clinical audit, maternal morbidity rate and blood transfusion rate in postpartum hemorrhage cases decreased significantly. This finding is in agreement with a multi-center research that instituting a protocol for the treatment of maternal hemorrhage significantly reduced the incidence of blood transfusion [14].

The present study showed a significant association between pre-eclampsia and ICU admission, which was similar with previous researches [12, 15]. In Lin’s study, obstetric ICU admission was nearly 4 times higher in women with hypertensive disorders in pregnancy [12]. Hitti et al. found that preeclampsia with severe features had the strongest association with severe maternal complications, followed by preeclampsia without severe features and chronic hypertension [15]. It is interesting that in the current study women with preeclampsia had over 7 folds higher risk for ICU admission, while chronic hypertension was not an independent risk factor. The reason for this disparity might be related to our referral system, where most women with chronic hypertension were referred to tertiary care in early pregnancy [16], whereas women with pre-eclampsia were often late-referrals with delayed diagnosis and suboptimal management [17]. This fact also implied that patients with uncomplicated hypertension could be safely managed in HDU, while preeclamptic patients with organ dysfunction frequently need intensive care. To reduce severe maternal complications related to preeclampsia, potential strategies include first trimester screening and prediction [18], low-dose aspirin prevention, early detection and tighter blood pressure control [19, 20].

### Generalizability and Limitations

The findings in this paper are based on a tertiary care, university-based hospital that cares for a higher percentage of medically complicated pregnancies. The high medical acuity of this population lends itself to a close evaluation of the changes in the characteristics of ICU admissions after introduction of obstetric HDU. A major limitation of our study is that it relies on a single center data. The outcomes would be fairly comparable with referral centers and may not be representative to population of our region. Therefore, it is possible that the increased trend in medical co-morbidities this study is caused by an increased number of referral and not a reflection of trend in the population. Moreover, there is no standardized criteria for HDU admission, which hampers comparison of data between reports and the interpretation of outcomes.

## Conclusions

Our study highlights the increasing role of HDU in management of severe maternal morbidities. Shifting of care for women with severe postpartum hemorrhage and hypertensive disorders in pregnancy to HDU may spare the ICU service for the more complex medical conditions. Whether providing intermediate care is cost-effective needs to be investigated in further studies.

## Data Availability

The datasets used and analyzed during the current study are available from the corresponding author on reasonable request.

## Abbreviations

HDU: High dependency unit
ICU: Intensive care unit
OR: Odds ratios
CI: Confidence intervals
aOR: adjusted odds ratios
BMI: Body mass index
